# Unravelling the mechanisms controlling heme supply and demand

**DOI:** 10.1073/pnas.2104008118

**Published:** 2021-05-25

**Authors:** Galvin C.-H. Leung, Simon S.-P. Fung, Andrea E. Gallio, Robert Blore, Dominic Alibhai, Emma L. Raven, Andrew J. Hudson

**Affiliations:** ^a^School of Chemistry, University of Leicester, LE1 7RH Leicester, United Kingdom;; ^b^Leicester Institute of Structural & Chemical Biology, University of Leicester, LE1 7HB Leicester, United Kingdom;; ^c^School of Chemistry, University of Bristol, BS8 1TS Bristol, United Kingdom;; ^d^Wolfson Bioimaging Facility, Faculty of Life Sciences, University of Bristol, BS8 1TD Bristol, United Kingdom

**Keywords:** heme biology, fluorescence lifetime imaging, biosensing

## Abstract

In addition to heme’s role as the prosthetic group buried inside many different proteins that are ubiquitous in biology, there is new evidence that heme has substantive roles in cellular signaling and regulation. This means that heme must be available in locations distant from its place of synthesis (mitochondria) in response to transient cellular demands. A longstanding question has been to establish the mechanisms that control the supply and demand for cellular heme. By fusing a monomeric heme-binding peroxidase (ascorbate peroxidase, mAPX) to a monomeric form of green-fluorescent protein (mEGFP), we have developed a heme sensor (mAPXmEGFP) that can respond to heme availability. By means of fluorescence lifetime imaging, this heme sensor can be used to quantify heme concentrations; values of the mean fluorescence lifetime (τ_Mean_) for mAPX-mEGFP are shown to be responsive to changes in free (unbound) heme concentration in cells. The results demonstrate that concentrations are typically limited to one molecule or less within cellular compartments. These miniscule amounts of free heme are consistent with a system that sequesters the heme and is able to buffer changes in heme availability while retaining the capability to mobilize heme when and where it is needed. We propose that this exchangeable supply of heme can operate using mechanisms for heme transfer that are analogous to classical ligand-exchange mechanisms. This exquisite control, in which heme is made available for transfer one molecule at a time, protects the cell against the toxic effect of excess heme and offers a simple mechanism for heme-dependent regulation in single-molecule steps.

## Significance

Heme is essential for the survival of virtually all living systems and is involved in many fundamental biological processes. It is also implicated as a signaling/regulatory molecule and must be mobilized in response to cellular demands. This presents a complex logistical problem: heme cannot simply diffuse around cells because it is both insoluble and cytotoxic. We show that the cell exhibits exquisite control over release of heme by limiting its availability to one molecule or less within cellular compartments. We suggest an exchange mechanism between protein partners to control supply and demand. Such a mechanism would provide an in-built buffering capacity for heme, enable cells to hoard supplies of heme, and protect the cell against the undesirable effects of heme.

Heme is essential for the survival of virtually all living systems—from bacteria, fungi, and yeast, through plants to animals. The family of heme proteins is vast, and heme proteins are responsible for a multitude of functions that are essential for the survival of the cell. To meet the needs of supply and demand for heme in cells, most organisms need to synthesize it. Biosynthesis of the heme cofactor is, therefore, one of the most important metabolic processes in biology; it occurs as an eight-step enzymatic pathway, the last three steps of which occur in the mitochondria (1). Surplus heme, on the other hand, is removed by heme oxygenase located in the endoplasmic reticulum (2). However, while the machinery for heme synthesis and degradation is well known, a decades-old question has been to establish precisely how heme is transported between its place of synthesis and subsequently made available to other regions of the cell where heme is in demand. Recent published work has hypothesized that membrane structures (3) and membrane contacts (4) are involved in the heme trafficking mechanism. Nevertheless, the scarcity of information in this area stands in stark contrast to the extensive efforts that have been directed toward understanding the structures and reactivities of many different heme proteins (e.g., refs. 5–8). An answer to this long-standing question on heme transport has recently become even more pressing because it is now established that heme has a regulatory/signaling role in the cell that goes well beyond the existing known requirements for heme in the housekeeping proteins that are essential for cell survival. These regulatory roles include transcriptional regulation and gas sensing, regulation of the circadian clock, and the gating of numerous ion channels (9–11). Deficiencies or excesses in cellular heme concentration also have widespread implications in health and disease [aging (12, 13), cardiovascular disease (14–16), inflammation (17–19), and immune response (17, 19–21)], and thus there is a need to understand the logistics of heme supply and demand.

The absolute requirement that heme, once synthesized, is then made available around the cell raises a number of fundamental questions that currently have no complete answers. One idea is that there is a pool of free heme to respond to cellular demands, and this has been discussed as far back as the 1970s (22, 23). However, the concept of a “heme pool” is problematic from both a chemical and biochemical perspective. The first is that heme is cytotoxic because it promotes the formation of free radicals through Fenton chemistry. So, if free heme is present in uncontrolled concentrations—for example, in a pool—then it would be a nuisance to the cell. A second problem is that heme is a hydrophobic molecule by virtue of its conjugated tetrapyrrole ring structure and is therefore insoluble; it also dimerizes extensively in aqueous solution (24) and in this form cannot be delivered to proteins that require only one molecule of heme per binding site. A free molecule of heme can therefore only exist transiently, and if a large reserve of heme is present, the heme molecules would presumably need to be exchanged rapidly between binding partners to remain solubilized, in the same way that heme is solubilized within the interior of other well-known heme proteins (e.g., hemoglobin). A third unknown is that while the need for availability of heme around the cell is undisputed with the exception of certain pathogenic bacteria [which do not synthesize heme and instead acquire it from the host (25)] and the CcmE chaperones for cytochrome *c* [in itself a special case as cytochrome *c* binds heme covalently (26, 27)], very few heme transporters have been identified (28–31).

New, more sensitive, and more sophisticated approaches are needed to develop a better understanding of the dynamics of cellular heme availability and the mechanisms that control it. Using fluorescence lifetime imaging (FLIM), we have probed the availability of heme in different locations of live cells via its interaction with a genetically encoded sensor. While extremely low concentrations of free heme have been determined quantitatively using this approach, the response of the sensor indicates the existence of a larger reserve of heme, which provides an exchangeable supply that can be mobilized between heme-binding partners. These results indicate that heme availability is not linked to a dedicated heme pool nor is controlled solely by specific heme chaperones. Instead, we propose that an exchangeable (buffered) reservoir of heme is present in the cell to provide not only a flexible supply of heme but also protection against the undesirable cytotoxic consequences of excess heme concentrations.

## Results

**Fig. 1.**
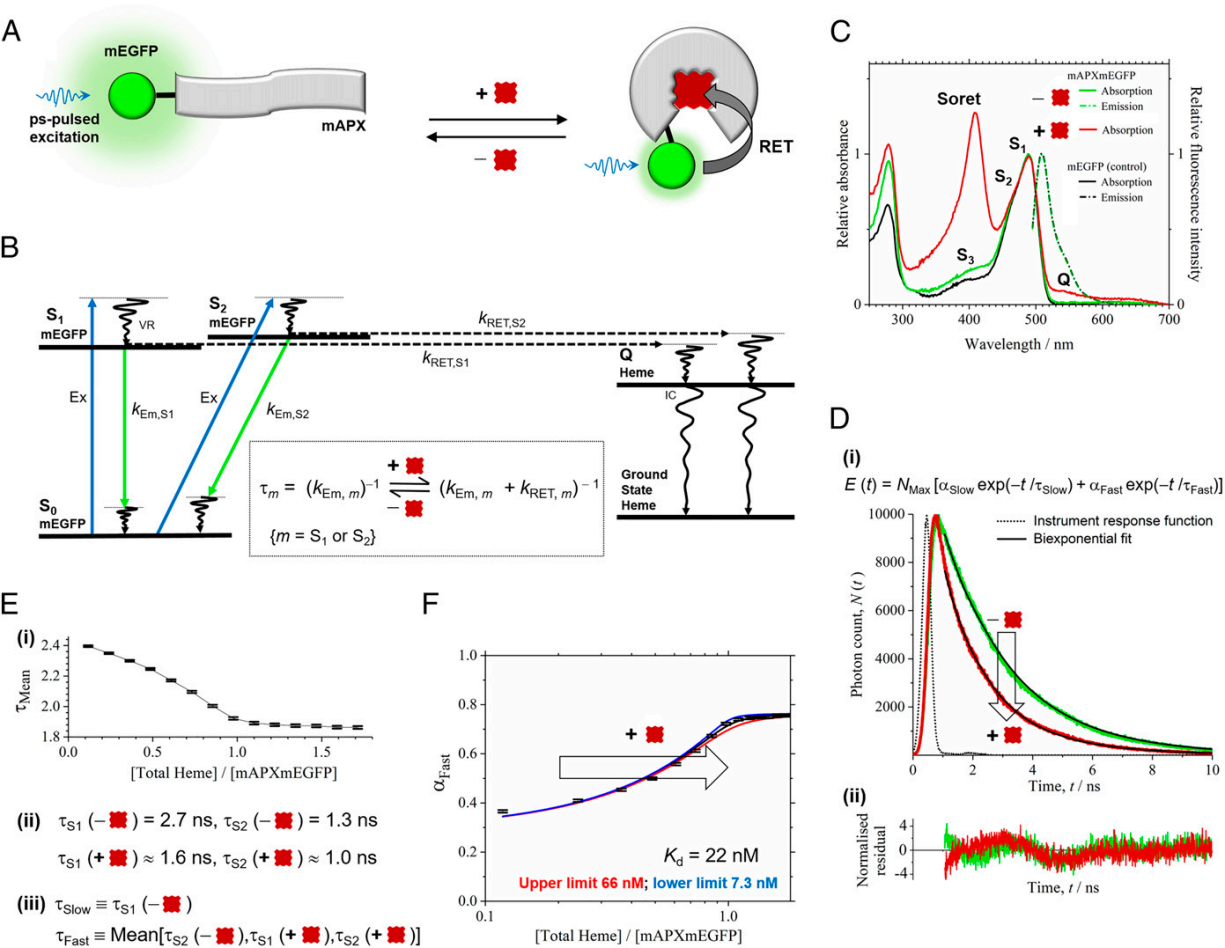
Heme sensing in cells. (*A*) The sensor is mAPX (gray) conjugated to mEGFP (green). Binding of heme (dark red) to mAPX results in RET from photoexcited mEGFP, with concomitant reduction in emission lifetime. (*B*) The principle of the sensor operation is based on different decay pathways from excited states of mEGFP in the presence and absence of heme. Excitation (Ex, blue) of mEGFP from the ground state, S_0_, to excited-vibronic states, S_1_ and S_2_, is followed by vibrational relaxation (VR). Decay pathways from S_1_ and S_2_ are either (*A*) fluorescence emission (green) with rate constants, *k*_Em,S1_ and *k*_Em__,__S2_, respectively, or (*B*) RET to an electronic-excited state of heme (c.f. mEGFP emission and heme Q band for overlap) with rate constants, *k*_RET__,__S1_ and *k*_RET__,__S2_, respectively, followed by VR and internal conversion (IC) to the ground state of heme. (*Inset*) Equations for fluorescence lifetimes, τ_S1_ and τ_S2_, in the presence and absence of heme. (*C*) Absorption and fluorescence spectra for mAPXmEGFP and mEGFP (λ_Ex_, 488 nm). Partially resolved bands (centered at 490 nm) for mEGFP are assigned to S_1_ and S_2_; a further weak absorption band for mEGFP (S_3_) and, on addition of heme, Soret and Q bands (408 and 541 nm) are observed. (*D*) (i) Time-correlated single-photon counting, *N* (*t*), from *apo*- (green) and *holo*-mAPXmEGFP (red) (λ_Ex_, 475 nm; λ_Em_, 510 nm; 37 °C) fitted to a biexponential-decay function, *E*(*t*), with time constants of 2.7 (τ_Slow_) and 1.3 ns (τ_Fast_). (ii) Normalized residuals for fitting to the decay profiles, (*N*(*t*) − *E*(*t*)) / √*E*(*t*). χ^2^ values were between 0.8 and 1.7 for biexponential fitting to the reported decay data. Significant improvement in χ^2^ cannot be achieved by inclusion of >2 decay terms. χ ^2^ = (1 / *h*) × Σ (*N* − *E*) ^2^/*E* {*h* time bins}. (*E*) (i) Sequential additions of heme to *apo*-mAPXmEGFP lead to a gradual increase in the amplitude of the fast (α_Fast_) relative to the slow (α_Slow_) component and a decrease in the mean lifetime, τ_Mean_. (ii) τ_S1_ and τ_S2_ for *apo*-mAPXmEGFP are the same as the optimized values of τ_Slow_ and τ_Fast_ from *E*(*t*). Both τ_S1_ and τ_S2_ are reduced in *holo*-mAPXmEGFP (see *B*, *Inset*), and estimates have been made for these values by assuming that *k*_RET__,__S1_ = *k*_RET__,__S2_ (*SI Appendix*). (iii) The biexponential model, *E*(*t*), for mixtures of *apo*- and *holo*- mAPXmEGFP has a slow-decay component, τ_Slow_, equal to τ_S1_ (*apo*) and a fast-decay component, τ_Fast_, equal to the mean of τ_S2_ (*apo*), τ_S1_ (*holo*), and τ_S2_ (*holo*). (*F*) A theoretical single-site binding model fitted to the amplitude, α_Fast_, in decay profiles obtained by sequential additions of heme to *apo*-mAPXmEGFP at 37 °C. The estimation of error bars in *E* and *F* is described in *SI Appendix*, section 2.

A heme sensor was constructed comprising the *apo* form (i.e., without heme) of a monomeric form of ascorbate peroxidase (APX), referred to here as *apo*-mAPX, fused to a monomeric form of green fluorescent protein (mEGFP) (as in Fig. 1*A*; further details of the construct designs are given in *Materials and Methods*). APX is a heme-dependent peroxidase (32). Its catalytic activity has been used for proteomic mapping in cells (33) and to identify the accumulation of heme in cellular compartments (34); EGFP has also been fused to APX in order to identify the cellular location of the enzyme (34). It is a homodimer in solution (34), and the monomeric (mutant) form was used in this work with mEGFP to avoid complexities arising from dimerization of either APX or GFP in the FLIM experiments below. This heme sensor (referred to as mAPXmEGFP) is able to quantify precisely heme concentration in live cells via measurement of the fluorescence lifetime of mEGFP. By recording the decay at a single emission wavelength, the ratio of *apo* to *holo* forms of mAPXmEGFP, and hence the heme concentration, can be determined precisely in our FLIM experiments. A major advantage of FLIM of mAPXmEGFP is that neither inner filtering of the fluorescence emission nor the partial inactivation of the fluorescent protein by photobleaching have an effect on photon emission times from mEGFP (35). Hence the two major limitations of previous designs for fluorescent heme sensors (4, 36–43) will not affect our experimental studies. Inner filtering and photobleaching does have a significant effect on the measured intensity of fluorescence emission, and all previous sensor designs comprising a heme-binding protein and one or more fluorescent proteins have relied on the measurement of fluorescent emission intensity to determine heme concentrations. In the work presented here, mAPXmEGFP uses a single fluorophore for lifetime studies; the decay parameters of mAPXmEGFP can be recorded accurately, and heme concentration can be determined more precisely.

Under irradiation from a picosecond-pulsed laser (at 475 to 488 nm), the fluorescence decay from *apo*-mAPXmEGFP contains two components derived from excitation to, and subsequently emission from, a pair of noninteracting excited states, S_1_ and S_2_ (Fig. 1 *B* and *C*) (44). The principle of the design is that binding of heme to the sensor facilitates resonance-energy transfer (RET; Fig. 1*B*) from these excited states of mEGFP to the heme chromophore and, subsequently, internal conversion and vibrational relaxation to the ground state. Hence, heme binding leads to a decrease in the emission lifetimes, τ_1_ and τ_2_, from each of the excited states of mEGFP (Fig. 1*B*, *Inset*).

In initial in vitro experiments, the *apo*-mAPXmEGFP protein was isolated and purified from *Escherichia coli* cultured in media supplemented with succinylacetone (*Materials and* *Methods*). Succinylacetone inhibits the second step of the eight-step heme synthesis pathway and thus limits the amount of heme available to the cells (45). mAPXmEGFP, expressed in *E. coli* under these growth conditions, is isolated 97% in the *apo* form (*Materials and Methods*). The absorption spectrum (green solid line in Fig. 1*C*) accordingly shows strong peaks only for mEGFP (i.e., 470 and 490 nm). On addition of heme to *apo*-mAPXmEGFP, a further absorption band is observed, as expected, in the heme Soret region (408 nm). Where mEGFP was expressed in *E.coli*, under the same conditions as for mAPXmEGFP (with the exception of the addition of succinylacetone), the absorption spectrum for mEGFP alone (black solid line in Fig. 1*C*) is similar to that for *apo*-mAPXmEGFP. The fluorescence emission spectrum of mEGFP is unchanged by fusion to mAPX (peak maximum at 509 nm; as shown in green and black dot-dash lines in Fig. 1*C*).

Fluorescence decay profiles for emission were measured for *apo*-mAPXmEGFP in the presence of varying amounts of heme and hence with different ratios of *apo* to *holo* (i.e., heme bound) forms of the sensor (Fig. 1*D*). The entire set of decay profiles (*n* = 14) can be fitted to a biexponential decay with fixed time constants of 2.7 (τ_Slow_) and 1.3 ns (τ_Fast_). The amplitude of the fast-decay component (α_Fast_) increases relative to the slow-decay component (α_Slow_) with sequential additions of heme leading to a decline in the intensity-weighted mean lifetime, τ_Mean_ (Fig. 1*E*, *i*; where τ_Mean_ = (Σ_*m*_ α_*m*_ × τ_*m*_^2^) / (Σ_*m*_ α_*m*_ × τ_*m*_) {*m* = Slow, Fast}; α_Slow_ + α_Fast_ = 1). The emission lifetimes τ_S1_ and τ_S2_ for *apo*- and *holo*-mAPXmEGFP correspond to the fitted time constants τ_Slow_ and τ_Fast_. Both τ_S1_ and τ_S2_ are reduced in *holo*-mAPXmEGFP compared to *apo*-mAPXmEGFP due to the competing nonradiative RET pathway (Fig. 1*B*, *Inset* and Fig. 1*E*, *ii*). Indirect evidence suggests that the emission spectra from S_1_ and S_2_ are near perfectly superimposed (*SI Appendix*, Fig. S1*B*). Because rate constants for RET depend on overlap between the emission spectrum of the donor and the absorption spectrum of the acceptor, *k*_RET,S1_ and *k*_RET__,__S2_, are expected to be approximately equal. Resonance energy transfer will make the values of τ_S1_ and τ_S2_ for *holo*-mAPXmEGFP higher and lower, respectively, than τ_S2_ for *apo*-mAPXmEGFP (*SI Appendix*, section 1 and Table S1). Hence it is possible to apply a biexponential decay model for all measurements containing different ratios of *apo*- and *holo*-mAPXmEGFP, where one of the components has a time constant (τ_Slow_) equal to the long decay lifetime (i.e., τ_S1_ in *apo*-mAPXmEGFP) and another component has a time constant (τ_Fast_) equal to the mean of the three short decay lifetimes (i.e., τ_S2_ in *apo*-mAPXmEGFP and both τ_S1_ and τ_S2_ in *holo*-mAPXmEGFP (Fig. 1*E*, *iii*)]. In this way, it is possible to determine the ratio of the concentrations of *apo*- to *holo*-mAPXmEGFP from the relative amplitudes of these fitted-exponential terms, α_Slow_ and α_Fast_ (*SI Appendix*, Eq. **S8**). A 1:1 binding model can be used to rationalize the changes in α_Slow_ and α_Fast_ observed following sequential additions of heme to *apo*-mAPXmEGFP (Fig. 1*F*, full details given in *SI Appendix*, section 2). The precision for the fitting of a 1:1 binding model to the lifetime data supports the proposed model given in Fig. 1*E*. Using this approach, the heme-dissociation constant for mAPXmEGFP has been determined (*K*_d_ = 22 nM; lower limit, 7 nM; upper limit, 66 nM). The *K*_d_ of mAPXmEGFP was found to be independent of both pH and ionic strength (*SI Appendix*, Fig. S3).

**Fig. 2.**
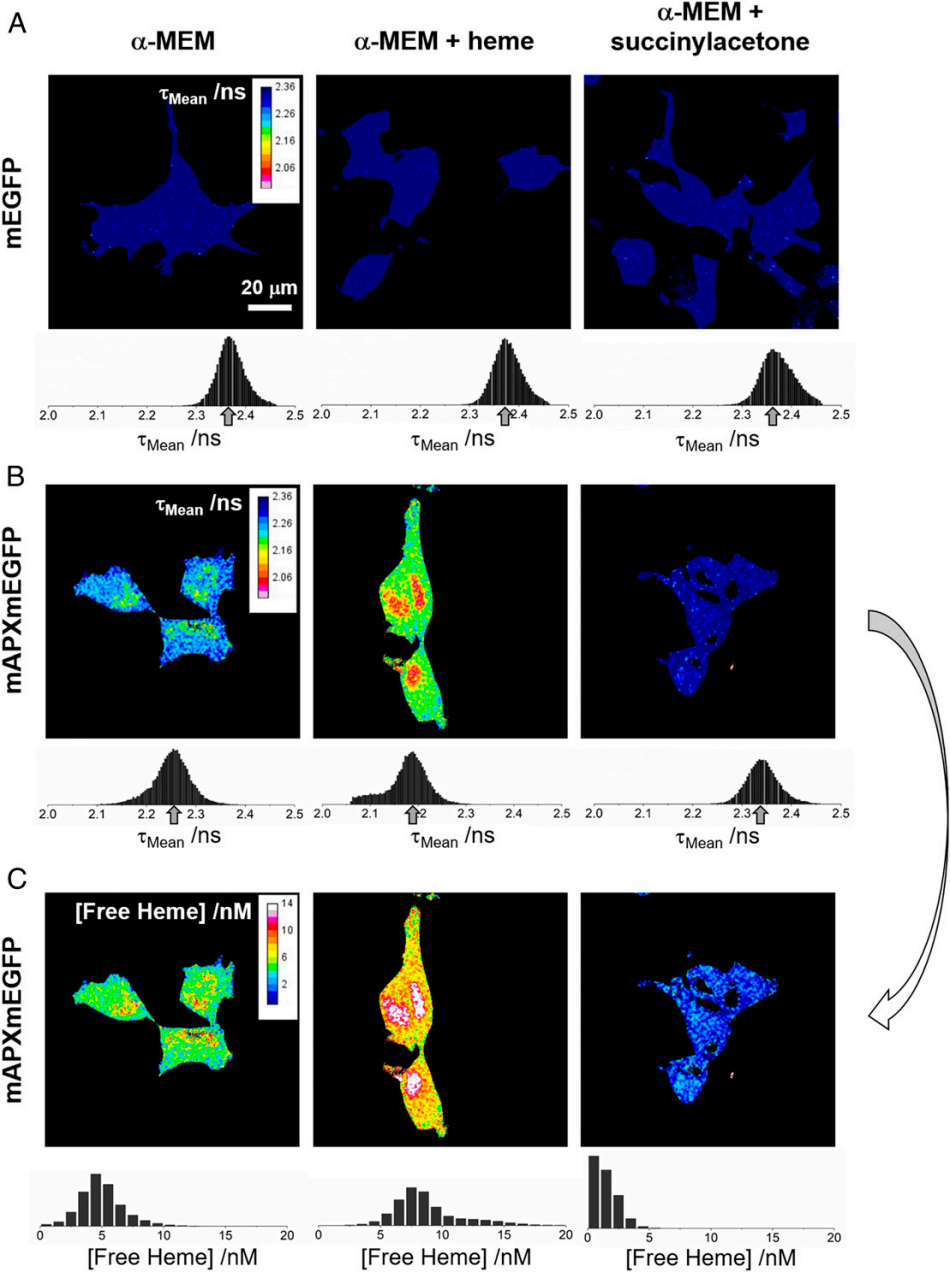
Color maps of intensity-weighted mean fluorescence lifetime and free heme concentration. The intensity-weighted mean fluorescence lifetime, τ_Mean_, for HEK293 cells expressing either (*A*) mEGFP alone or (*B*) mAPXmEGFP and cultured under different conditions (λ_Ex_, 488 nm; λ_Em_ > 495 nm). (*Left*) α-MEM (with 10% fetal bovine serum); (*Middle*) α-MEM supplemented with heme (10 μM; 24 h prior to imaging); (*Right*) α-MEM depleted of heme following addition of succinylacetone (1 mM; 24 h prior to imaging). If the photon count was below a value of 200 for an individual pixel, then a black color was assigned to that pixel in the color map (this is the threshold criteria used in the FLIM experiments for the estimation of the decay parameters; *Materials and Methods*). Each of the color maps in *A* and *B* are accompanied by a histogram showing the frequencies for which particular values of τ_Mean_ occur in the spatial distribution of pixels for the images of cells under different conditions. An arrow indicates the modal pixel value of τ_Mean_. (*C*) The concentration of free heme calculated from the relative amplitudes, α_Slow_ and α_Fast_, in the images shown in *B* using the heme-dissociation constant, *K*_d_, for mAPXmEGFP determined in Fig. 1*F* (22 nM at 37 °C). The full calculation is outlined in *SI Appendix*, section 3. The detection limit for [Free Heme] is <1 nM (0.6 molecule per fL). Each of the color maps for free heme concentration is accompanied by a histogram showing the frequencies for which particular values of [Free Heme] occur in the spatial distribution of pixels for the images of cells under different conditions. The black color has been used to represent pixels for which the photon count was below the threshold of 200. All colors other than black represent reliable measurements of [Free Heme]. For the images in the *Left* column of *A* and *B*, the time-correlated single-photon counting data and the fitted-biexponential decay curves are shown in *SI Appendix*, Fig. S6.

Subsequent to these in vitro experiments above, both mEGFP (alone) and *apo*-mAPXmEGFP were expressed in separate HEK293 cell lines. The total concentration of expressed mAPXmEGFP was estimated to be ca. 1 μM (*SI Appendix*, Fig. S4). Expression of mEGFP in HEK cells allows α_Slow_ and α_Fast_ to be determined in the absence of RET (as the latter requires heme in close proximity to mEGFP, Fig. 1*B*). The fluorescence decay was measured at different locations in HEK cells by confocal FLIM. The measured decays were fitted to separate biexponential functions, and the resulting amplitudes and lifetimes of the decay components were used to calculate intensity-weighted mean lifetimes, τ_Mean_ = (Σ_*m*_ α_*m*_ × τ_*m*_^2^) / (Σ_*m*_ α_*m*_ × τ_*m*_) {*m* = Slow, Fast}. Fig. 2 shows color maps of τ_Mean_ for cells expressing both mEGFP (alone) and *apo*-mAPXmEGFP. Each of the color maps is accompanied by a histogram showing the frequencies for which particular values of τ_Mean_ occur in the spatial distribution of pixels for the images of cells under different conditions. While the concentration of the sensor is highest in the cytosol, there is still a sufficient concentration of the sensor in the nucleus and other parts of the cell. In the presence of high concentrations of free heme, there is substantial quenching of the sensor fluorescence in the nucleus (*SI Appendix*, Fig. S6). The time constants obtained for a biexponential function using a global-fitting algorithm to the imaging data for both mEGFP and mAPXmEGFP were 2.5 (τ_Slow_) and 1.2 ns (τ_Fast_). Fluorescence lifetimes for the sensor were expected to be marginally shorter in HEK cells (in vivo) than those observed for the purified protein (in vitro; Fig. 1*D*) due to the inverse dependence of fluorescence lifetime on the square of the refractive index (27, 29). The average χ^2^ value for biexponential modeling of the pixel data in a single image was 0.99 (*SI Appendix*, Fig. S6 and section 6).

For both mEGFP and mAPXmEGFP, separate images were obtained for HEK293 cells grown in α-minimum essential medium (α-MEM; Fig. 2, *Left*), in α-MEM supplemented with heme (Fig. 2, *Middle*), and in α-MEM supplemented with succinylacetone to deplete heme from cells (Fig. 2, *Right*). For mEGFP, the modal value for τ_Mean_ does not change in the images recorded under the three different conditions—this is evident as the peak in the histogram is between 2.36 and 2.37 ns (Fig. 2*A*, *Left*, *Middle*, and *Right* ). In each example for mEGFP, the distribution of τ_Mean_ is approximately symmetrical with a narrow full-width half-maximum of 0.06 to 0.07 ns. In contrast, for mAPXmEGFP, the modal value for τ_Mean_ does change significantly in the images recorded under the three different cell culture conditions. In α-MEM, the modal value for τ_Mean_ of mAPXmEGFP was 2.25 ns (Fig. 2*B*, *Left*), which is significantly lower than the value measured for mEGFP above due to RET from the excited mEGFP chromophore within the subpopulation of mAPXmEGFP that contains a bound molecule of heme (refer to Fig. 1*B*). Under heme-supplemented conditions, the modal value of τ_mean_ was further reduced to 2.19 ns (Fig. 2*B*, *Middle*). There is a considerable tail in the distribution toward shorter lifetimes, which indicates that there are regions of the cell with much higher concentrations of heme. Under heme-depleted conditions, the modal value for τ_Mean_ of mAPXmEGP was 2.33 ns (Fig. 2*B*, *Right*), which is close to that observed in the cell lines expressing mEGFP alone (Fig. 2*A*). This is consistent with there being no heme bound to mAPXmEGP under these heme-depleted conditions. All of the images shown in Fig. 2 contain multiple cells (>2, up to 5 cells). The modal value and the spatial distribution of values for τ_Mean_ is consistent among the discrete numbers of cells in each image recorded under the different conditions.

These FLIM experiments show that values of τ_Mean_ are lowered in mAPXmEGFP when a heme molecule bound to mAPX is located in close proximity to mEGFP (<10-nm distance). In the case of the HEK cell lines expressing mEGFP on its own (Fig. 2*A*), the absence of a heme-binding domain fused to the fluorescent protein means that there is little variation in τ_Mean_ even when there are significant changes in cellular concentrations of heme. In contrast, for the HEK cells lines expressing mAPXmEGFP (Fig. 2*B*), there are substantive differences in modal values of τ_Mean_ measured under conditions of different heme concentration. Using the results from the in vitro studies (Fig. 1), it is possible to transform the imaging data from live cells (Fig. 2*B*) to construct a map illustrating the concentration of free heme in the cell (Fig. 2*C*; *SI Appendix*, Eqs. **S17** and **S18**—the full calculation is described in *SI Appendix*, section 3). Free heme will be a small fraction of the total heme present in cells. There will be considerably larger fractions associated with known hemoproteins and bound reversibly to other heme-binding partners. In free heme, the iron ion exists in a square planar complex with protoporphyrin IX only (the axial coordinate positions could be occupied by water molecules). Each of the color maps for free heme concentration, in Fig. 2*C*, are accompanied by a histogram showing the frequencies for which particular values of concentration exist in the spatial distribution of pixels for the images of cells under different conditions. The breadth of the distribution shown in the histogram plots indicate how the spatial distribution of free heme varies within the discrete number of cells in each of the images. The free heme concentration determined here has a modal value of 4 to 5 nM under normal conditions, reducing to <1 nM under heme-depleted conditions. These concentrations correspond to 2.4 to 3.0 molecules of heme per fL (control conditions) to <1.0 molecule per fL (under heme-depleted conditions).

**Fig. 3.**
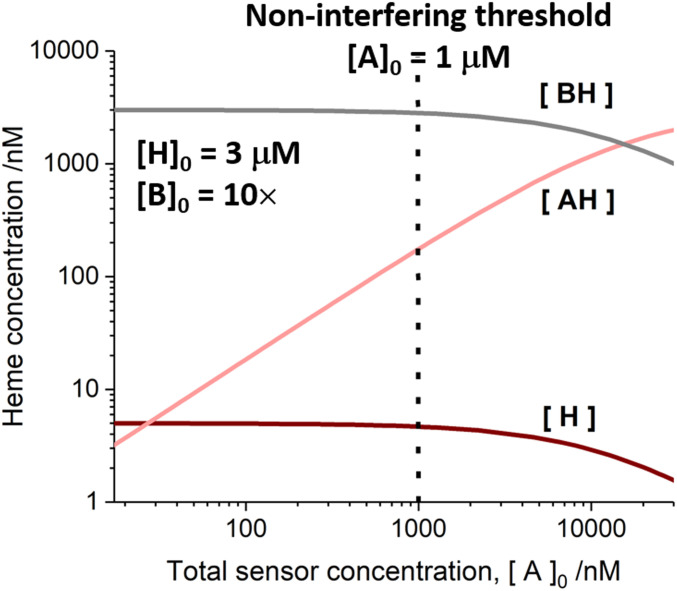
A computational model illustrating how the concentration of exchangeable heme varies as a function of the total concentration of the expressed sensor, [A]_0_; A = mAPXmEGFP. Exchangeable heme will be mostly associated with binding partners, B (where [BH], in gray, represents the concentration of heme bound to B). A minute proportion of the exchangeable heme exists as free molecules, H (with the concentration, [H], shown in dark red). At higher values of [A]_0_, a larger proportion of exchangeable heme will be transferred to the sensor. The concentration of the *holo* form of the heme sensor, [AH], is shown in light red), where the total concentration of the expressed sensor, [A]_0_ = [A] + [AH] is given on the horizontal axis ([A] = concentration of the *apo* form of the sensor). The model has been created by assuming that the total concentration of heme, [H]_0_ = [H] + [BH] + [AH], is 3 μM (46); the concentration of free heme, [H], in the absence of a sensor or acceptor protein is 5 nM (as observed in Fig. 2, *Lower Left*); and the *K*_d_ for heme dissociation from the sensor is 22 nM (Fig. 1*F*). The total concentration of heme-binding partners, [B]_0_ = [B] + [BH], has been assumed to be 10 × [H]_0_ to achieve effective buffering of heme concentration. We have determined an experimental value of 1 μM for the cellular concentration of the sensor (dotted line, *SI Appendix*, Fig. S4). At and below these concentrations, the expression of the sensor does not interfere with the availability of heme in cells, and the concentration of exchangeable heme (both [BH] and [H]) is unaffected, as shown in the plot.

We have explored the possibility that the presence of the sensor can perturb the concentration of free heme in cells. A model has been constructed, in which the sensor (A = mAPXmEGFP in Fig. 3) competes with the *apo*-proteins (B in Fig. 3) for the available heme in the cell. At the measured expression level for the sensor of ca. 1 μM (*SI Appendix*, Fig. S4), the sensor will not perturb the cellular biochemistry by depleting the availability of heme (forming a complex AH) as long as the total concentration of heme exceeds or equals 3 μM (the value reported in ref. 46 from denatured cell lysates). In this model, the total concentration of heme will include a minute fraction of free heme (estimated to be 5 nM; as in Fig. 2*C*, *Left*) and a larger fraction associated with reversible binding partners, BH.

## Discussion

The quantitative maps of free heme concentration derived from the FLIM experiments demonstrate very low amounts of free heme (ca. 5 nM). We interpret this to mean that free heme represents a minute fraction of the entire amount of heme present in the cell. Indeed, this concentration of free heme stands in sharp contrast to the much higher concentration of 3 μM measured in ref. 46 following the denaturing of cell lysates; this much higher concentration would be dominated by contributions from the population of heme molecules that are bound to proteins rather than the small amounts of free heme. Our in vivo measurement of free heme in HEK293 cells is also one to two orders of magnitude lower than the reported values in other cell lines: HeLa (42), yeast (41), and IMR90 (11). These authors have described the concentrations as representing labile heme (41, 42) and regulatory heme (11) but, in these earlier publications, the exact identity of the heme species in labile heme and regulatory heme has not been described.

Numerous substantive conclusions derive from this quantitative measurement reported in Fig. 2. Perhaps the most obvious is that the long-held concept of a pool of free heme (22, 23) becomes immediately irrelevant because there are too few molecules of heme available to support a dedicated pool. By restricting free heme to such miniscule concentrations, the problems associated with heme-dependent cytotoxicity are also solved because the probability of free heme reacting with oxygen or other reactive oxygen species is much reduced.

Thinking more widely, these minute and presumably transient concentrations of free heme will be dwarfed by the total heme complement within the cell, which will be incorporated into multiple housekeeping and other proteins that are essential for cellular function. For a proportion of this total heme complement, heme binding to a respective protein is so tight as to be practically irreversible—with the heme only released upon enzymatic degradation and not available for movement around the cell or for interaction with the sensor in our experiments. However, for the experiments in Fig. 2*B* to be viable, at least some proportion of the total heme complement—that which is not bound irreversibly to housekeeping proteins—must be available for exchange. Our experiments are thus consistent with the idea that there is a population of the total heme complement that is bound more weakly and therefore reversibly to heme-binding partner proteins or to other molecules (which might include free amino acids) that can buffer against changes in the heme concentration. The changes in concentration of free heme observed in the imaging experiments are relatively small (<0.6 to 2.4 molecules per fL; Fig. 2*C*), which is consistent with a buffering mechanism. These heme molecules that are weakly bound to buffer molecules, along with the miniscule population of free heme, would constitute a body of exchangeable heme in the cell.

**Fig. 4.**
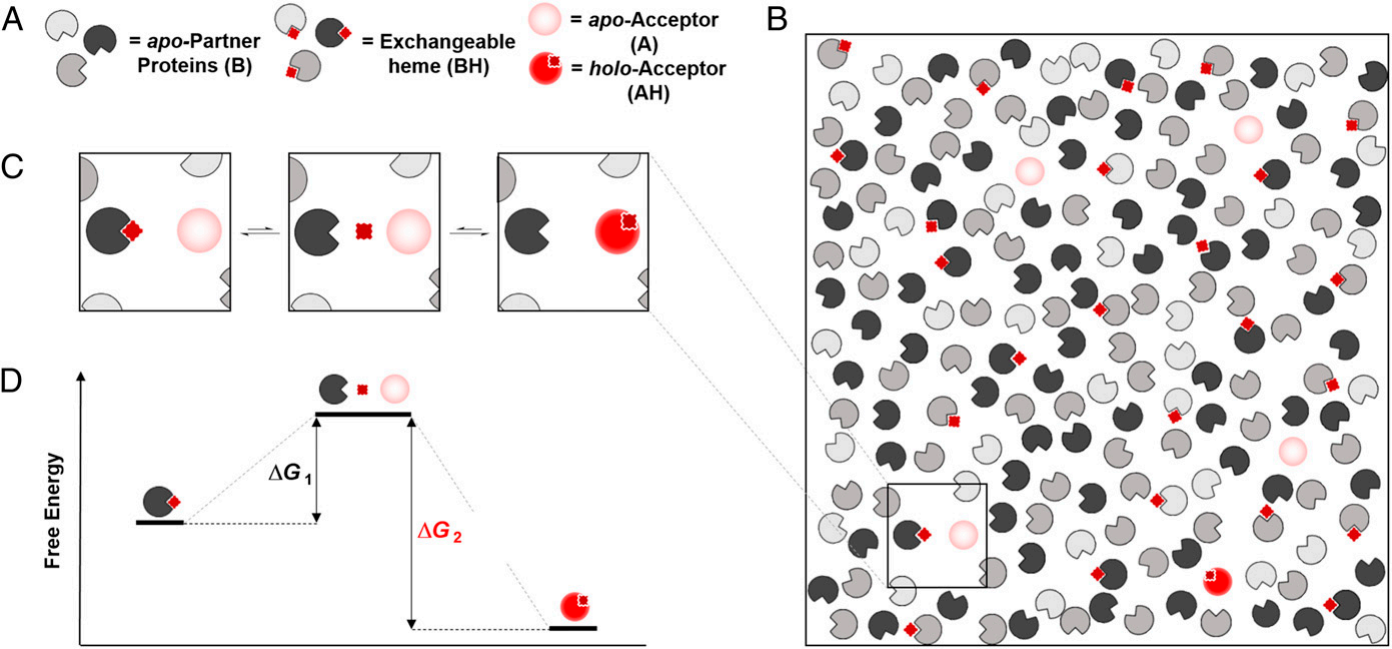
An exchange mechanism for managing heme supply and demand in cells. (*A* and *B*) We envisage a body of reversible heme-binding partners, represented by pacmans in different shades of gray (referred to as B in Fig. 3), along with potential heme-acceptor proteins (shaded light red pacmans; these might also be the sensor, referred to as A in Fig. 3). These heme-binding partners could be known heme proteins [indoleamine 2,3-dioxygenase, for example, is known to lose its heme under certain conditions (47)] or other proteins such as GAPDH (28, 29, 31). Heme is shown as a dark red square or diamond (referred to as H in Fig. 3). (*C*) Heme exchanges between heme-binding partners via dynamic chemical equilibria. (*Left*) Heme in the cell is bound to a heme-binding protein: we refer to this as exchangeable heme. (*Middle*) Dissociation of heme from a heme protein is typically a rare event; it results in an *apo*-partner protein and a molecule of free heme. These free heme molecules are presumed to be transiently formed and may be ligated with weak, easily dissociated ligands (e.g., H_2_O) or otherwise solvated (24). (*Right*) The heme thus released then binds to other heme-acceptor proteins (light red) (or is picked up by a sensor). The free heme reported by our sensors (Fig. 2) measures the likelihood that heme will dissociate from a partner protein, with higher concentrations describing a more frequent event. Thus, the free heme in each region of a cell is controlled by the dissociation constants and the relative abundance of all heme-binding partner proteins in that region. (*D*) A consequence of the thermodynamic reversibility of heme binding is that the transfer of heme can be facilitated between the exchangeable pool and another protein. The energy profile of this heme exchange mechanism is indicated.

A model that is consistent with the sensor measurements is outlined in mechanistic form in Fig. 4. The foundation for the model is an exchange mechanism for managing heme supply and demand in cells involving a body of reversible heme-binding partners, which might be known heme proteins or other proteins (see legend). In earlier publications, the existence of what was termed a regulatory heme pool (48, 49) or intracellular heme pool (50) was proposed to account for varied observations in blood disorders such as porphyria (51, 52), gene expression (22, 53, 54), heme protein levels (23), and heme biosynthesis (55, 56). More recently, the weakly bound fraction of the total heme complement has been referred to as labile heme (41, 42, 57) and regulatory heme (11). While the model presented in Fig. 4 can still be reconciled with earlier work, it uses the concept of exchangeable heme, as introduced above, to account for both the presence of weakly bound heme (to act as a buffer against changes in heme concentration) and the transient existence of free heme (which is measured precisely by the FLIM experiment). The model in Fig. 4 also describes a mechanism for heme exchange. We see clear advantages of such an exchange mechanism between protein partners, designed for the purpose of managing heme supply and demand. It would provide a powerful buffering capacity for the cell to mitigate changes in heme concentration. This heme-buffering capacity would be useful to manage changes in the supply and demand of heme that require immediate adjustment and that cannot be mitigated in a timely manner by up-regulation of heme oxygenase or heme synthesis. A supply of exchangeable heme, to be made available on demand in this highly controlled fashion as shown in Fig. 4 *A* and *B*, could also be utilized for precise and tightly regulated heme-dependent signaling control.

This process of heme exchange as described above is analogous to classical mechanisms for ligand and solvent exchange that have long been established in transition metal chemistry (58). Exchange of heme between partners in cells is simple in concept, as outlined in Fig. 4, but by analogy with the versatile ligand exchange mechanisms that are known in metal complexes could conceivably proceed by several different routes (e.g., associative, dissociative, or interchange mechanisms). It would avoid the need for specific heme chaperones which, aside from exceptional examples [such as GAPDH (31)], have persistently eluded identification over decades. Note, however, that our data do not rule out the use of chaperones altogether, and they might be useful in mammalian systems in cases where more directed heme transfers or allocations are required. We expect that a cell will take advantage of all possible mechanisms for transfer of heme from the exchangeable reservoir, but one example is illustrated in Fig. 4*C*. A dissociative pathway, as shown in Fig. 4*C*, provides a mechanism by which the release of at least a single molecule of heme has a finite probability. Even transient release of a free molecule of heme into the aqueous medium of the cell would be sufficient for its capture by another protein (such as a regulatory or signaling heme protein) or, as in this work, by a sensor. Free heme concentrations in the region of 1 molecule per fL, as identified here, in the vicinity of a signaling site are still sufficient to ensure its population with heme by this exchange process, although dissociation of heme might present an activation energy barrier (Fig. 4*D*). The concentration of free heme is controlled by the relative rates of association and dissociation of heme from reversible-binding partners. While the former process dominates leading to only a few molecules of free heme per fL (or a transient existence for an individual molecule of free heme), the binding partners have sequestered a much larger concentration of exchangeable heme. We speculate that the total concentration of exchangeable heme will remain relatively constant in the cell; however, the transiency of free heme (i.e., the free heme concentration) could be controlled by changes in the relative composition of the buffering ensemble, which is expected to differ in various compartments of the cell to meet specific local demands for heme. An alternative mechanism might involve association of exchange partners [as suggested for exchange of metal ions in cells (59), *SI Appendix*, Fig. S7].

In the mechanism shown in Fig. 4*C*, the abundance of heme-binding partners dictates both the overall buffering capacity and hence the free heme concentration in the cell (as shown in *SI Appendix*, Fig. S5*A*). Fixing the availability of heme within a defined range allows the cell to exclusively supply heme to proteins possessing a *K*_d_ value below the threshold of free heme concentration which is thus generated (this concept is illustrated in *SI Appendix*, Fig. S5*B* using a simple competitive binding model). This exquisite control also provides a mechanism for heme-dependent signaling and regulation, as heme can be supplied discretely, leading to the switching on of proteins in single-molecule steps. Since changes in the availability of heme could be deleterious due to switching on/off of heme dependent functions, the control of heme supply is vital to the cell.

Armed with a better understanding of heme localization and mobilization across compartments in cells, the future holds the possibility to establish how deficiencies or excesses of heme affect the regulation of numerous functions known to have heme dependencies [e.g., regulatory proteins involved in gene expression and circadian response (60–62)]. Our model of heme availability will lead to a more complete understanding of the activities and consequences of these regulatory proteins and various immune pathologies for which heme homeostasis is a key driver (20). Since heme concentrations are known to increase during hypoxia and after thrombosis/stroke, there are important consequences for cardiovascular disease (14), as well as in neuronal survival and aging which are also dependent on heme (12).

## Materials and Methods

### Protein Expression and Purification for In Vitro Characterization of mEGFP and mAPXmEGFP.

mAPX was created from the wild-type protein by incorporation of the K14D and E112K mutations and then expressed recombinantly as a fusion protein with mEGFP (63) in a pLEICS-45 vector carrying ampicillin resistance and a N-terminal His tag. This fusion protein—containing the double mutation (K14D/E112K)—is referred to as mAPXmEGFP in this work. mEGFP was inserted into a pLEICS-01 vector carrying ampicillin resistance and a N-terminal His tag. For in vitro characterization experiments, mEGFP alone and the mAPXmEGFP sensor were expressed in *E. coli* BL21(DE3). In both cases, cells were grown in lysogeny broth (LB) at 37 °C until the optical density at 600 nm was 0.6 to 1.0. Protein expression was induced with 250 μM isopropyl-β-D-thio-galactoside and incubated at 23 °C overnight. For expression of mAPXmEGFP in *E. coli*, the LB media was supplemented with succinylacetone (1 mM; Sigma Aldrich); otherwise, the protocols for expression of mAPXmEGFP and mEGFP were the same. Cells were pelleted by centrifugation (3,000 *g*, 30 min, 4 °C) and resuspended in a buffer solution containing 10 mM potassium phosphate (pH = 7) and 150 mM KCl followed by addition of lysozyme (2 mg/mL; Sigma Aldrich), DNase (0.1 mg/mL; Sigma Aldrich), and protease inhibitor mixture (Roche). The solution was then sonicated on ice in cycles of 30 s (on)/ 30 s (off) for 30 min. The lysate was clarified by centrifugation (20,000 *g*, 30 min, 4 °C). Both mEGFP and mAPXmEGFP were purified from the supernatant by loading onto a nickel affinity column (Ni-NTA Agarose, Qiagen), washing with 100 mM imidazole, and elution with 300 mM imidazole. The eluate was desalted with a PD10-G25 (GE Healthcare) column and further purified on a HiLoad Superdex 200-pg column (GE Healthcare) in 10 mM phosphate buffered saline (pH = 7) containing 150 mM KCl (64). The concentration of the protein was estimated using an absorption coefficient for EGFP of 53,000 M^−1^ ⋅ cm^−1^ at 514 nm (65). The absorption coefficient for *holo*-APX is 107,000 M^−1^ ⋅ cm^−1^ at 410 nm (66). Using this value to estimate the amount of *holo*-mAPXmEGFP, the percentage of protein isolated in the *apo* form (97%; see main text) was determined.

Others have reported on the multimeric nature of APX (67) and EGFP (68). By utilizing monomeric forms of these proteins and avoiding the possibility of the fused construct oligomerizing through association of either heme-binding or fluorescent protein domains, a precise quantitative measurement of heme via fluorescence lifetime is possible. If one or both of the protein domains dimerizes in an APX + GFP construct, there will exist alternative pathways for nonradiative energy transfer between the fluorescent protein if there is more than one bound heme molecule. This will have an impact on the number of decay components and the intensity-weighted mean lifetime for the fluorescence emission. Hence, a monomeric sensor is essential to ensure a 1:1 stoichiometric interaction between the reporter and heme for quantitative mapping via lifetime measurements of heme concentrations in live cells. The sensor used in our experiments comprises the monomeric forms of both the heme-binding protein (the K14D/E112K variant of APX, referred to as mAPX), and mEGFP.

### In Vitro Time-Correlated Single-Photon Counting of Fluorescence Emission from mEGFP and mAPXmEGFP.

Time-correlated single-photon counting decay curves measured from in vitro protein samples (1 μM concentration of protein, in 10 mM potassium phosphate (pH = 7)), were measured using a Horiba Jobin Yvon fluorimeter (Fluorolog & Fluorohub) with pulsed laser excitation (478 nm; <200-ns pulse duration). Data sets of decay profiles were fitted globally to a multiexponential function using the FLIMfit software (69). Unless otherwise stated, all measurements were made at 37 °C.

### Mammalian Cell Transfection.

HEK293 cells purchased from the European Collection of Authenticated Cell Cultures were maintained in α-MEM (Gibco) supplemented with 10% fetal bovine serum (Gibco) at 37 °C and 5% CO_2_. Cells were transfected with mAPXmEGFP in pLEICS-138 vector or mEGFP in pLEICS-12. Transfection was performed with Lipofectamine 3000 reagent (Thermo Fischer Scientific) according with the manufacturer’s protocol. Stable cell lines were generated by selection with 500 µg/mL Geneticin (G418, Thermo Fischer Scientific) over a period of 3 wk. A Western blot was performed on lysates to estimate the concentration of mAPXmEGFP from HEK293–pLEICS-138–mAPX cells using known quantities of recombinant protein spiked into wild-type HEK293 as a protein standard (*SI Appendix*, Fig. S4).

### FLIM of Mammalian Cell Lines.

Cells were seeded into CELLview dishes (Greiner Bio-One) and were grown in phenol red–free α-MEM media supplemented with 10% fetal bovine serum. Separate compartments on cell dishes were treated 24 h prior to imaging with either 1 mM succinylacetone or 10 µM heme (iron protoporphyrin IX chloride, hemin). FLIM was performed on a laser-scanning confocal microscope equipped with a pulsed white light laser (Leica SP8X; University of Bristol, Wolfson Bioimaging Facility). An excitation filter was used with a band pass centered at 488 nm, and fluorescence emission was collected between 495 and 550 nm. Cells were maintained at 37 °C, with 5% CO_2_. A global-fitting algorithm for multiexponential models was applied to analyze all the pixel decay profiles in images recorded from cell lines expressing mEGFP or mAPXmEGFP (69). Individual values for the amplitudes and lifetimes of the decay components were used for both the calculation of free heme concentration (*SI Appendix*, section 3) and the calculation of the intensity-weighed mean lifetime, τ_mean_, in order to generate color maps of the heme distribution in cells (see *Results*). If the photon count was below 200 for a single pixel in an image, then a black color was assigned to that pixel in the color maps. Any assigned color other than black reports a reliable value for τ_mean_; a photon count of 200 was the threshold criteria in the FLIM experiments for the estimation of the decay parameters. Even in spatial locations of the cell in which the concentration of the sensor is low (i.e., in the nucleus, where photon counts were between 200 and 500), a consistent measurement of τ_mean_ was obtained across the pixels in these regions of a cell (see the white and red areas in Fig. 2*B*). As long as reliable and consistent values for the decay parameters could be determined, then it has been possible to report values for the concentration of free heme.

## Supplementary Material

Supplementary File

## Data Availability

All study data are included in the article and/or *SI Appendix*.

## Change History

June 11, 2021: The license for this article has been updated.
