# Supplementary material for: Unravelling the mechanisms controlling heme supply and demand
Source: Proc Natl Acad Sci U S A. 2021 May 25;118(22):e2104008118. doi: 10.1073/pnas.2104008118 (PMC8179208; doi:10.1073/pnas.2104008118)
Supplement: Supplementary File [file pnas.2104008118.sapp.pdf]

PNAS
www.pnas.org

Supplementary Information for

Unravelling the mechanisms controlling heme supply and demand

Galvin C-H. Leung[1,2], Simon S.-P. Fung[1,2], Andrea E. Gallio[3], Robert Blore[1,2], Dominic Alibhai[4], Emma L. Raven[3]* and Andrew J. Hudson[1,2]*

[1]School of Chemistry, University of Leicester, Leicester, United Kingdom, LE1 7RH.

[2]Leicester Institute of Structural & Chemical Biology, University of Leicester, Leicester, United Kingdom, LE1 7RH.

[3]School of Chemistry, University of Bristol, Cantock's Close, Bristol, United Kingdom, BS8 1TS.

[4]Wolfson Bioimaging Facility, Faculty of Life Sciences, Biomedical Sciences Building, University of Bristol, University Walk, Bristol, BS8 1TD

* Emma L. Raven and Andrew J. Hudson.

Email: emma.raven@bristol.ac.uk; andrew.hudson@leicester.ac.uk.

Emma L. Raven ORCID 0000-0002-1643-8694
Andrew J. Hudson ORCID 0000-0003-1849-9666

This PDF file includes:

Supplementary text
Figures S1 to S7
Tables S1
Appendix

Supplementary Information Text

1. The relationship between (a) the fraction of mAPXmEGFP that contains a bound molecule of heme and (b) the optimised values of α_{Slow} and α_{Fast} from the fluorescence decay.

Time constants of 2.7 ns (τ_{Slow}) and 1.3 ns (τ_{Fast}) were obtained for biexponential fitting to TCSPC decay profiles measured from *in vitro* mixtures of *apo*- (heme free) and *holo*- (heme bound) mAPXmEGFP (see Fig. 1 D; in these measurements, the purified protein was expressed and isolated from *E.coli*). The values of α_{Slow} and α_{Fast} in the biexponential-decay function are dependent on the relative amounts present of *apo*- and *holo*-mAPXmEGFP.

The fluorescent protein, mEGFP, can be excited to either of two non-interacting excited states, S_1 and S_2 (see Fig. 1 (B) main). The fluorescence lifetimes, τ_{S1} and τ_{S2}, are altered when heme binds to mAPXmEGFP (see Fig. 1 (B) inset) due to the competition between resonance energy transfer and fluorescence emission.

In *apo*-mAPXmEGFP, the emission lifetime of S_1 will be longer than that of S_2. The lifetime values, τ_{S1} (*apo*) and τ_{S2} (*apo*), can be reconciled with the time constants, τ_{Slow} = 2.7 ns, and τ_{Fast} = 1.3 ns, respectively. Both τ_{S1} (*holo*) and τ_{S2} (*holo*) will be reduced relative to the corresponding values for *apo*-mAPXmEGFP due to competing non-radiative resonance-energy transfer (RET) pathways (see Fig. 1 (B) inset). Assuming $k_{RET,S1}$ is equal to $k_{RET,S2}$, then the effect of RET is to make the values of τ_{S1} (*holo*) and τ_{S2} (*holo*) higher than and lower than 1.3 ns, respectively; for example, if $k_{RET,S1}$ and $k_{RET,S2}$ are both equal to 2.5×10^8 s^{-1}, then τ_{S1} (*holo*) = 1.6 ns and τ_{S2} (*holo*) = 1.0 ns. The small differences between the values of τ_{S2} (*apo*) = 1.3 ns, τ_{S1} (*holo*) =1.6 ns and τ_{S2} (*holo*) =1.0 ns means that the individual lifetimes cannot be resolved in the fluorescence decay profiles and, hence, the emission from S_2 (*apo*), S_1 (*holo*) and S_2 (*holo*) contribute together to the second decay component in the fitted-biexponential function with time constant, τ_{Fast}. This time constant is thus best expressed as the mean of the corresponding lifetimes of the three contributing states:

$$\tau_{Slow} = \tau_{S1}(apo) = 2.7 \text{ ns} \quad [1]$$

$$\tau_{Fast} = \text{Mean}\{\tau_{S2}(apo), \tau_{S1}(holo), \tau_{S2}(holo)\} = 1.3 \text{ ns} \quad [2]$$

Measured and estimated values for all the photophysical parameters defined in Fig. 1 (C) are given in Table S1.

Following irradiation with light in the range 470 – 490 nm, ε is defined as the probability for photoexcitation of mAPXmEGFP to S_1 (hence, 1–ε will be the probability for photoexcitation to S_2). f is the fraction of mAPXmEGFP with a bound molecule of heme bound; and Q_{S1} and Q_{S2} are the quantum yields for fluorescence from S_1 and S_2, respectively. The values of Q_{S1} and Q_{S2} are altered when heme binds to mAPXmEGFP:

$$Q_{S1}(apo) = k_{Em,S1}\tau_{Slow} \times \varepsilon \times (1 - f) \quad [3]$$

$$Q_{S2}(apo) = k_{Em,S2}\tau_{Fast} \times (1 - \varepsilon) \times (1 - f) \quad [4]$$

$$Q_{S1}(holo) = k_{Em,S1}\tau_{Fast} \times \varepsilon \times f \quad [5]$$

$$Q_{S2}(holo) = k_{Em,S2}\tau_{Fast} \times (1 - \varepsilon) \times f \quad [6]$$

The integrated intensites for emission contributing to the observed slow and fast decay components are α_{Slow} τ_{Slow} and α_{Fast} τ_{Fast}, respectively. Hence,

$$\frac{\alpha_{\mathrm{Fast}}\tau_{\mathrm{Fast}}}{\alpha_{\mathrm{Slow}}\tau_{\mathrm{Slow}}} = \frac{Q_{S2}(apo) + Q_{S1}(holo) + Q_{S2}(holo)}{Q_{S1}(apo)} \qquad [7]$$

The following equations are obtained by substituting equations [3] – [6] into [7]:

$$\frac{\alpha_{\mathrm{Fast}}}{\alpha_{\mathrm{Slow}} + \alpha_{\mathrm{Fast}}} = \frac{f + C}{1 + C} \quad \text{where} \quad C = \frac{k_{\mathrm{Em,S1}}}{k_{\mathrm{Em,S2}}}\left(\frac{1}{\varepsilon} - 1\right) \qquad [8]$$

Equation [8] provides the relationship between (a) the fraction of *holo*-mAPXmEGFP, *f*, and (b) the optimised values, α_{Slow} and α_{Fast}, from analysis of the fluorescence decay.

2. Fitting a theoretical single-site binding model to the optimised values, α_{Slow} and α_{Fast}, from analysis of fluorescence decays following sequential additions of heme to *apo*-mAPXmEGFP.

TCSPC decay profiles (*n* = 14) were obtained following sequential additions of a heme stock solution (0.2 mM iron protoporphyrin IX chloride, hemin, in 10 mM potassium phosphate) to an *in vitro* purified sample of mAPXmEGFP (see Results). By utilising a single-site binding model, customised fitting software (see Appendix to the Supplementary Information, SI) predicted the fraction of *holo*-mAPXmEGFP that was present following each addition of titrant, *f*(*i*), and the constant term, *C*, in equation [8]. Predicted values of *f*(*i*) and *C* were obtained by minimising χ^2 between experimental and calculated values of $\alpha_{\mathrm{Fast}}(i) / (\alpha_{\mathrm{Slow}}(i) + \alpha_{\mathrm{Fast}}(i))$ using a simplex algorithm to iteratively improve the fitting of the binding model:

$$\chi^2 = \frac{1}{m}\sum_{i=1}^{n}\left(\left(\frac{\alpha_{\mathrm{Fast}}(i)}{\alpha_{\mathrm{Slow}}(i) + \alpha_{\mathrm{Fast}}(i)}\right)_{Experimental} - \left(\frac{f(i) + C}{1 + C}\right)_{Binding\ model}\right)^2 \qquad [9]$$

where *m* is the number of degrees of freedom. The binding is described by the equilibrium and the dissociation constant, K_{d}, below, where $c_{free\ heme}(i)$, $c_{apo}(i)$ and $c_{holo}(i)$ are the concentrations of free heme and *apo*- and *holo*- mAPXmEGFP, respectively.

$$holo\text{-mAPXmEGFP} \rightleftharpoons apo\text{-mAPXmEGFP} + \text{free heme} \qquad [10]$$

$$K_{\mathrm{d}} = \frac{c_{apo}(i) \times c_{free\ heme}(i)}{c_{holo}(i)} \qquad [11]$$

If p_0 is the concentration of protein in the original sample, p_0 is the concentration of the heme titrant, V_0 is the initial sample volume, and $\delta V(i)$ is the total volume of titrant added, then the concentrations of free heme and *apo*-mAPXmEGFP are:

$$c_{apo}(i) = p_0 \times \frac{V_0}{V_0 + \delta V(i)} - c_{holo}(i) \qquad [12]$$

$$c_{free\ heme}(i) = h_0 \times \frac{\delta V(i)}{V_0 + \delta V(i)} - c_{holo}(i) \qquad [13]$$

Substitution of [12] and [13] into [11] gives the quadratic equation:

$$0 = c_{holo}(i)^2 - \left(\frac{p_0 V_0 + h_0 \delta V(i)}{V_0 + \delta V(i)} + K_\mathrm{d}\right) c_{holo}(i) + \frac{p_0 V_0 h_0 \delta V(i)}{\left(V_0 + \delta V(i)\right)^2} \qquad [14]$$

The parameters, K_d, and p_0 were varied by the simplex algorithm, and the quadratic equation in [14] solved for each addition of titrant (i = 1, n) to obtain values for $c_{holo}(i)$ which were then used to calculate values of $f(i)$:

$$f(i) = \frac{c_{holo}(i)}{p_0 \times \frac{\delta V(i)}{V_0 + \delta V(i)}} \qquad [15]$$

The photoexcitation probability, ε, was also varied by the simplex algorithm and calculated values of α_{Fast} (i) / (α_{Slow} (i) + α_{Fast} (i)) (in accord with the theoretical binding model) were obtained using equation [8]. These calculated values were compared with the experimental values using the χ^2 formula in equation [9]; and the iterative algorithm was terminated when a minimum value of the χ^2 obtained.

This calculation provided an estimate of the best-fit value of K_d to the fluorescence decay measurements for mixtures of *apo*- and *holo*-mAPXmEGFP. The analytical fit was further refined by accounting for the presence of a small concentration of heme in the initial protein sample; mAPXmEGFP was estimated to be isolated 97% in the *apo*- form (see Methods). The fitting program used to estimate K_d is outlined in Appendix to the SI.

The precision of the analytical fit has been evaluated by taking into consideration the error bars on the values for of α_{Fast}. The error bars have been estimated using the following method. The data points in fluorescence decay curves (such as those shown in Fig. 1D) exhibit photon noise obeying Poisson statistics. Other contributions to signal noise (for example, read noise of the photon counter) will be negligible in comparison to photon noise. The decay curves in Fig. 1D were obtained by time-stamping photons incident on an avalanche photodiode, and counting the number of photons in time bins with a width of 6.95 ps. The measurement was halted when the population in one of the time bins reached 10 000 photons. Similar decay curves have been simulated using a computer program, written in Fortran 90, with parameters identical to the experiments (6.95ps time bins, 10 000 photon count). The calculated decay was a biexponential function with time constants of 2.7 and 1.3 ns, as in Fig. 1D; and the relative amplitudes, α_{slow} and α_{fast}, were varied in order to create theoretical decay curves for each of the experimental data points in Fig. 1E and 1F. The calculated decay profiles were transformed by generating a random deviate from a Poisson distribution for each of the data points; hence, the theoretical data were transformed into a noisy-decay profile realistic of experimental data.

For each pair of relative amplitudes, α_{slow} and α_{fast}, a total of 10 replicates for the decay profiles were calculated. These data were analysed using the same global-fitting algorithm applied to the experimental decay profiles ((*1*); see Methods), and the reported values for the amplitudes of a fitted biexponential curve were compared with the actual values used to create the noisy-decay profiles. The standard deviation of the amplitudes obtained from the fitted functions have been used to estimate the error bars in Fig. 1E and 1F.

By plotting binding curves corresponding to K_d values larger and smaller than the optimised value, the precision of the analytical fit could be determined by estimating the range of K_d that produce curves passing through the error bars.

3. Calculating free heme concentrations from fluorescence lifetime images of mAPXmEGFP in live cells.

The normalised amplitude for the fast decay component of the fluorescence emission of mAPXmEGFP in each of the pixels, with coordinates (*i*, *j*), in 2D lifetime images of live cells is given by a formula equivalent to equation [8]:

$$\frac{\alpha_{\mathrm{Fast}}(i,j)}{\alpha_{\mathrm{Slow}}(i,j)+\alpha_{\mathrm{Fast}}(i,j)}=\frac{f(i,j)+C}{1+C}\quad \text{where}\ C=\frac{k_{\mathrm{Em,\,S_1}}}{k_{\mathrm{Em,\,S_2}}}\left(\frac{1}{\varepsilon}-1\right)\quad [16]$$

The value for *C* in equation [16] can be estimated from the mean value of α_{Fast} / (α_{Slow} + α_{Fast}) determined across all the pixels in images of cells expressing mEGFP alone. The fluorescence protein, mEGFP, which cannot bind heme, provides a measurement of α_{Fast} / (α_{Slow} + α_{Fast}) for *f* is equal to 0 (in this case, equation [16] can be rearranged to find the value of *C*). From the images in Fig. 2 A, the estimated value of *C* is 0.264. The *in vivo* photophysical parameters for mAPXmEGFP (*i.e.* in HEK293 cells) differ from the corresponding *in vitro* parameters for the purified protein, given in Table S1; these include the rate constants, k_{Em} and k_{RET}, and lifetimes, τ. The reasons for the differences are likely a combination of factors including variation in the refractive index and rotational diffusional coefficient between solution and cellular environments. Hence, it was necessary to prepare a cell line in which mEGFP alone is expressed, in addition to the cell line in which mAPXmEGFP is expressed, in order to obtain a baseline measurement in which mEGFP emission is not perturbed by the presence of heme.

When a value of *C* has been determined from images of mEGFP alone, the fraction of *holo*-mAPXmEGFP, *f* (*i*, *j*), can be estimated in subsequent images (see Fig. 2 B):

$$f(i,j)=\frac{\alpha_{\mathrm{Fast}}(i,j)}{\alpha_{\mathrm{Slow}}(i,j)+\alpha_{\mathrm{Fast}}(i,j)}(1+C)-C \qquad [17]$$

Using the value of K_d determined from experiments using the purified protein, mAPXmEGFP, the concentration of free heme can be determined from values of *f* (*i*, *j*):

$$c_{free\ heme}(i,j)=\frac{K_{\mathrm{d}}}{\frac{1}{f(i,j)}-1} \qquad [18]$$

Equation [18] was used to calculate the colour maps of free heme concentration in HEK 293 cells shown in Fig. 2 C.

4. Variation of the decay parameters for mEGFP in different environments.

Recombinant mEGFP was studied *in vitro* to characterise how the relative amplitudes, α_{Slow} and α_{Fast}, and time constants, τ_{Slow} and τ_{Fast}, for biexponential fitting to fluorescence decay profiles depend on pH, temperature, ionic strength, refractive index and emission wavelength (the excitation wavelength is fixed in these experiments; λ_{Ex} = 475 nm).

Both α_{Slow} and α_{Fast} and τ_{Slow} and τ_{Fast} were pH independent between 5.5 and 8.0, and insensitive to the ionic strength and emission wavelength (λ_{em}) between 510 to 560 nm (see Fig. S1). The decay times, τ_{Slow} and τ_{Fast}, were dependent on temperature and refractive index only (see Fig. S2).

The insensitivity of α_{Slow} and α_{Fast} (along with τ_{Slow} and τ_{Fast}) to the emission wavelength is significant. For mEGFP, in the absence of RET pathways, the separate lifetime components can be assigned to emission from the S_1 and S_2 states. The absence of any significant variation in α_{Slow} and α_{Fast} indicates that the individual spectra corresponding to emission from S_1 and S_2 must be near-perfectly superimposed. Since a rate constant for RET will depend on the overlap between the emission spectrum from a mEGFP state and the absorption spectrum for the Q state of heme, then the rate constants, $k_{RET,S1}$ and $k_{RET,S2}$, must be similar.

We anticipated that the decay parameters observed for *in vitro* experiments using purified mAPXmEGFP might differ from those measured *in vivo* from mAPXmEGFP in HEK 293 cells. This could be due to variation in pH, ionic strength and refractive index between the solution and cellular environments. The time constants, τ_{Slow} and τ_{Fast}, determined for biexponential fitting to the fluorescence decay of mAPXmEGFP in HEK 293 cells (*in vivo*) were slightly lower than those observed for the purified protein *in vitro* (2.5 and 1.2 ns, *vs.* 2.7 and 1.3 ns). The *in vitro* experiments utilised a laser centred at 475 nm for excitation of mEGFP (λ_{ex}) and the fluorescence lifetime images were recorded using excitation centred at 488 nm. A different excitation wavelength is expected to contribute to differences in measured values of α_{Slow} and α_{Fast} because the initial population of S_1 and S_2 states will be different (the photoexcitation probability to S_1, ε, will be different). The *in vitro* measurements, using a shorter λ_{Ex}, resulted in higher relative values of α_{Fast} (c.f. Fig. 1 F).

The time constants, τ_{Slow} and τ_{Fast}, determined for biexponential fitting to the fluorescence decay of mEGFP alone were found to be statistically similar to those obtained for mAPXmEGFP in the *in vivo* measurements in HEK 293 cells; however, the values of τ_{Slow} and τ_{Fast} were slightly longer for mEGFP than mAPXmEGFP when the measurements were made *in vitro* (c.f. Figs. S2 and 1 D).

5. *In vitro* measurement of the heme dissociation constant, K_d , for the sensor under different conditions.

By altering the pH and ionic strength of the solution, we have explored conditions that could change the structure of mAPXmEGFP and, hence, potentially change the heme-dissociation constant, K_d. There is no significant variation in K_d with ionic strength and pH; see Fig. S3.

6. Validation of different decay models for fitting to fluorescence lifetime data for mAPXmEGFP in live cells.

In the fluorescence lifetimes images of mAPXmEGFP in HEK 293 cells (Fig. 2), we have fitted the decay data to a biexponential model despite the potential existence of a total of 4 components, i.e. apo- and holo- forms of mAPXmEGFP excited to either the S1 or S2 states. The rationale is that the time constants for emission from the S2 state of apo-mAPXmEGFP and from both the S1 or S2 states of holo-mAPXmEGFP are sufficiently close that they cannot be resolved by fitting a 4-exponential model. Likely reasons for the proximity of these time constants are given in 2.1 and 2.4. We have attempted to fit a 4-exponential model using a global-fitting algorithm to the fluorescence lifetime imaging data both (i) by allowing the 4 time constants to be optimized, and (ii) by fixing the time constants commensurate with fluorescence emission from apo-mAPXmEGFP (i.e. 2.5 and 1.2 ns), and allowing a pair of time constants to be optimised for emission from holo-mAPXmEGFP. Neither method resulted in a significantly improvement in the χ^2-value for the analytical fit to the decay data, nor identified additional time constants between 500 ns and 3μs (see Fig. S6).

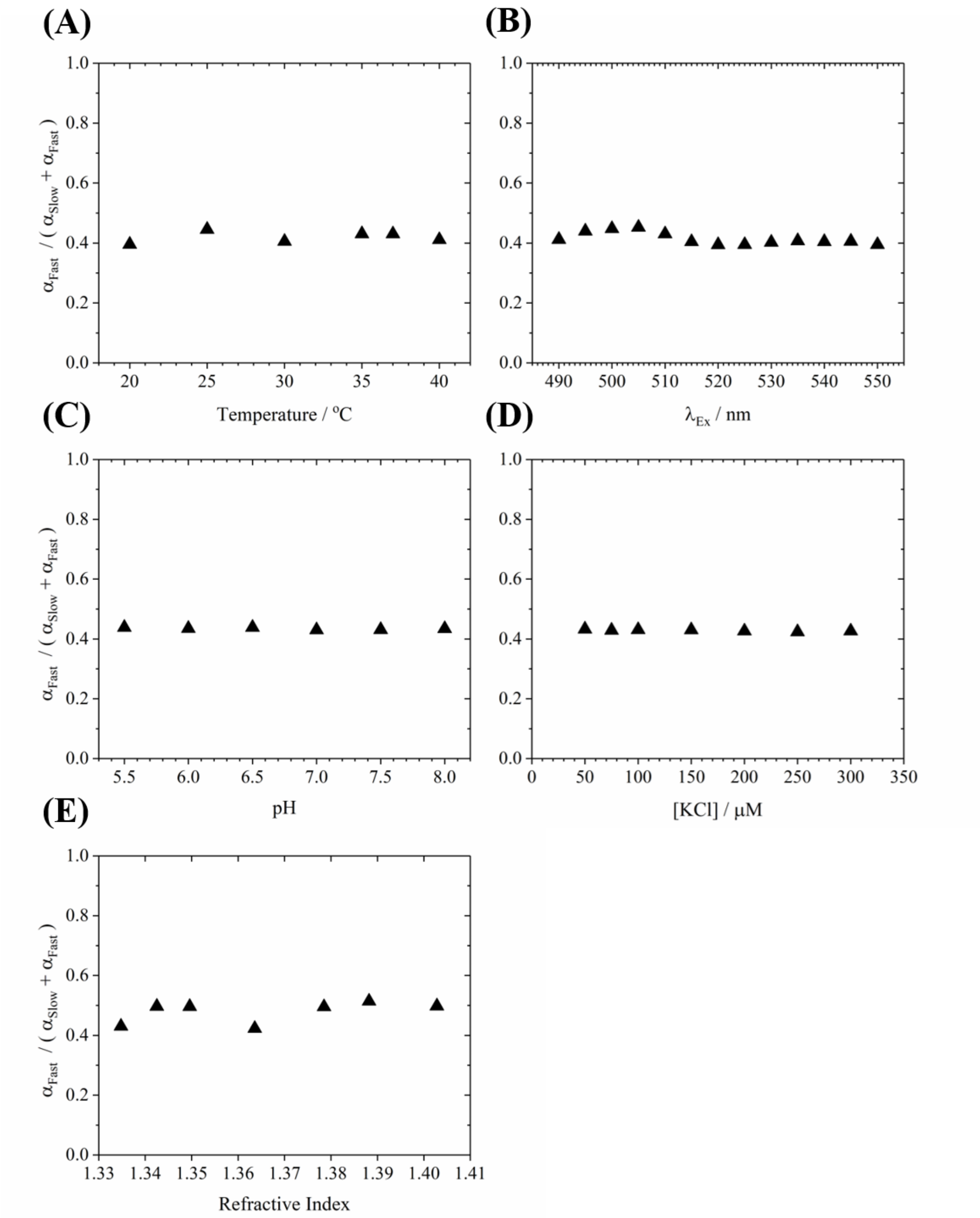

Fig. S1. The normalised amplitude of the fast component in the fluorescence decay as a function of **(A)** temperature, **(B)** emission wavelength, **(C)** pH, **(D)** ionic strength and **(E)** refractive index. There is almost no variation with these parameters for different experimental conditions or cellular environments. However, the decay times vary for differences in refractive index and temperature (see Figure S2).

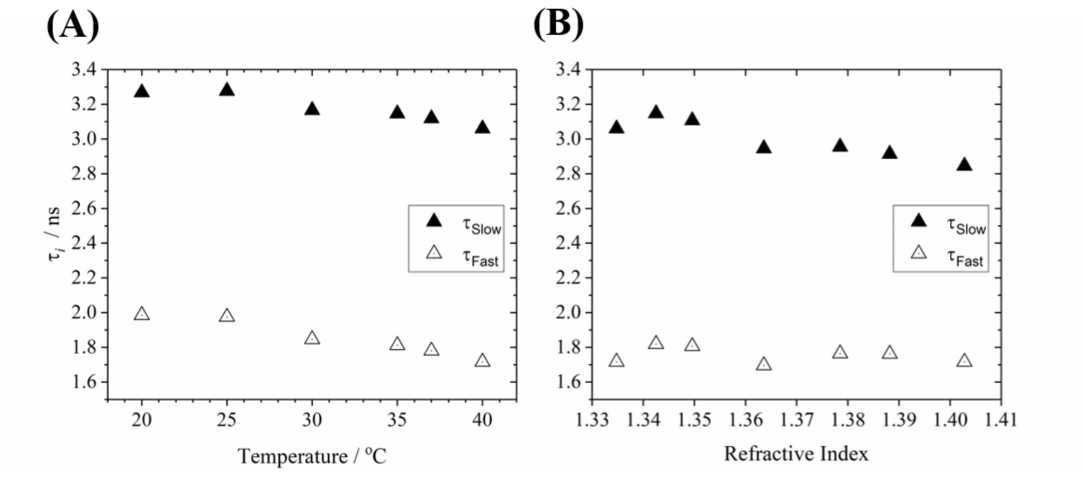

Fig. S2. The decay times for the slow and fast components of the fluorescence emission as a function of **(A)** temperature and **(B)** refractive index.

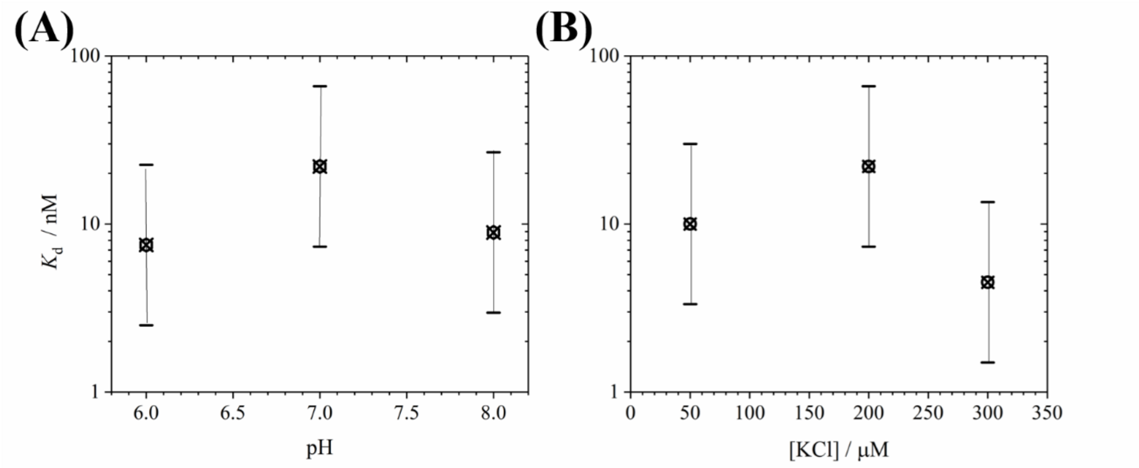

Fig. S3. The heme-dissociation constant, K_d, of mAPXmEGFP as a function of **(A)** pH, and **(B)** ionic strength. The magnitude of the error bars is explained in Section 2 and in Fig. 1F.

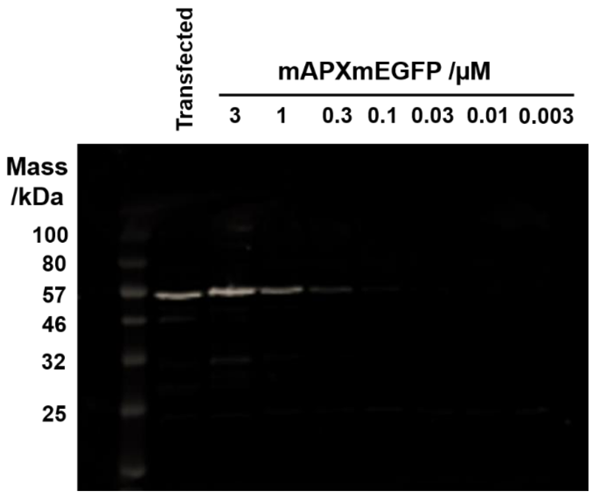

Fig. S4. Quantification of mAPXmEGFP in HEK293 cells by western blot. Expression of mAPXmEGFP in the stably transfected HEK293 cell line was quantified by detection on a western blot with an anti-GFP antibody (GF28R, #MA5-15256, Invitrogen) and comparing this level of expression with known concentrations of recombinant mAPXmEGFP (corresponding to 0.003 to 3 µM) that were spiked into wildtype HEK293 lysates. The intensity of the mAPXmEGFP band (55 KDa) was found to be closest to the 1 µM mAPXmEGFP protein standard. There is negligible free mEGFP in transfected cells suggesting that the sensor is stable. The average volume of HEK293 cells was assumed to be 3 pL.

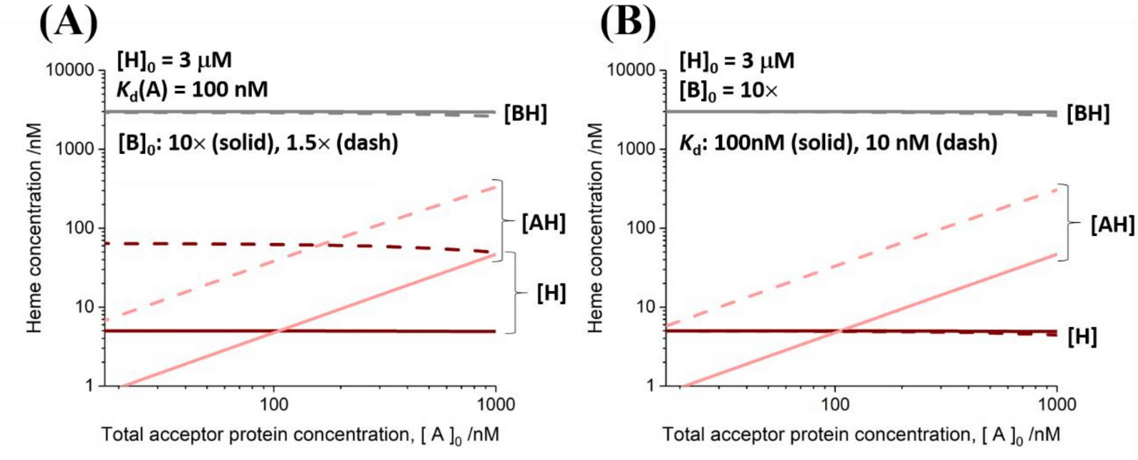

Fig. S5. A computational model illustrating how the concentration of exchangeable heme varies as a function of the total concentration of an acceptor protein, $[A]_0$. Exchangeable heme will be mostly associated with binding partners, B (where [BH], in grey, represents the concentration of heme bound to B). A minute proportion of the exchangeable heme exists as free molecules, H (with the concentration, [H], shown in dark red). At higher values of $[A]_0$, a larger proportion of exchangeable heme will be transferred to the acceptor protein. The concentration of the *holo*-form of the acceptor protein, [AH], is shown in light red, where the total concentration of the acceptor protein, $[A]_0 = [A] + [AH]$ is given on the horizontal axis ([A] = concentration of the *apo* form of the acceptor). The model has been created by assuming that the total concentration of heme, $[H]_0 = [H] + [BH] + [AH]$, is 3 μM (*2*); the concentration of free heme, [H], in the absence of an acceptor protein is 5 nM (as observed in Fig. 2 (lower left)). **(A)** A model for how the cell could either increase or decrease transfer of exchangeable heme to an acceptor protein by altering the concentration of heme-binding partners, $[B]_0$, and hence the buffering capacity of the cell (where $[B]_0 = [B] + [BH]$). In this example, the concentration, $[B]_0$, is reduced from $10\times[H]_0$ to $1.5\times[H]_0$, and the effect of this change is an increase in the concentration of free heme, [H], and an increase in the concentration of the *holo*-form of an example of an acceptor protein, [AH] (with K_d 100 nM). **(B)** When the concentration of free heme, [H], is 5 nM, the availability of heme is such that the cell supplies heme exclusively to acceptor proteins possessing a K_d value of the same order of magnitude or below 5 nM (*i.e.*, acceptor proteins with K_d 1 nM or 10 nM).

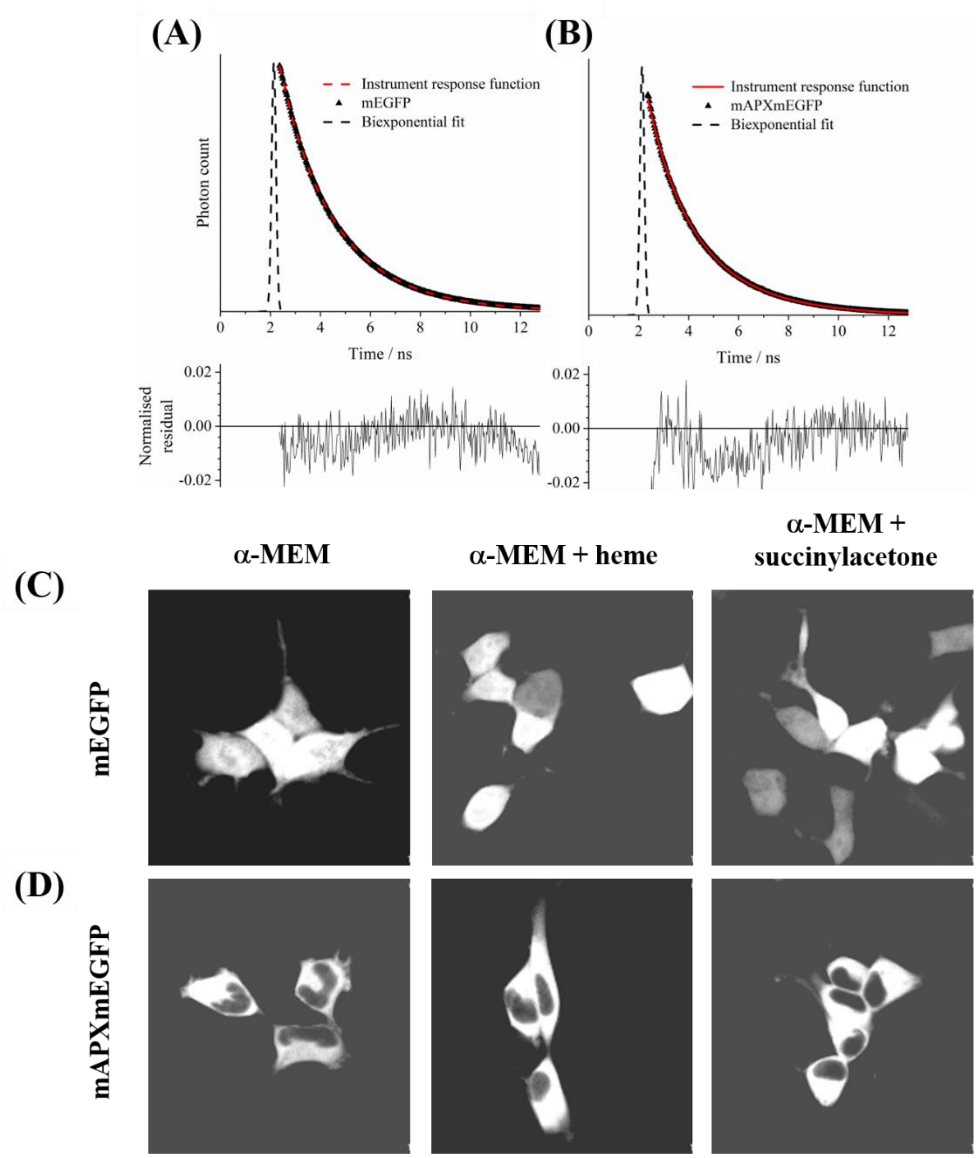

Fig. S6. Time correlated single photon counting decay curves for **(A)** mEGFP and **(B)** mAPXmEGFP in HEK 293 cells (λ_{Ex}, 488 nm). The cumulative photon count and normalised residual is shown for the entire image shown in Fig. 2 A (left) and B (left) in (untreated) α-MEM. The χ^2 value for a biexponential fit to the mEGFP image is 1.23; and the χ^2 value for a biexponential fit to the mAPXmEGFP image is 0.92. Fluorescence intensity images for HEK293 cells expressing either (**C**) mEGFP alone or (**D**) mAPXmEGFP and cultured under different conditions (λ_{Ex}, 488 nm; λ_{Em} > 495 nm). (**Left Column**) α-minimum essential medium (α-MEM; with 10% foetal bovine serum); (**Middle Column**) α-MEM supplemented with heme (10 μM; 24 h prior to imaging); (**Right Column**) α-MEM depleted of heme following addition of succinylacetone (1 mM; 24 h prior to imaging). The fluorescence intensity images correspond to the lifetime images shown in Fig. 2 (A) and (B).

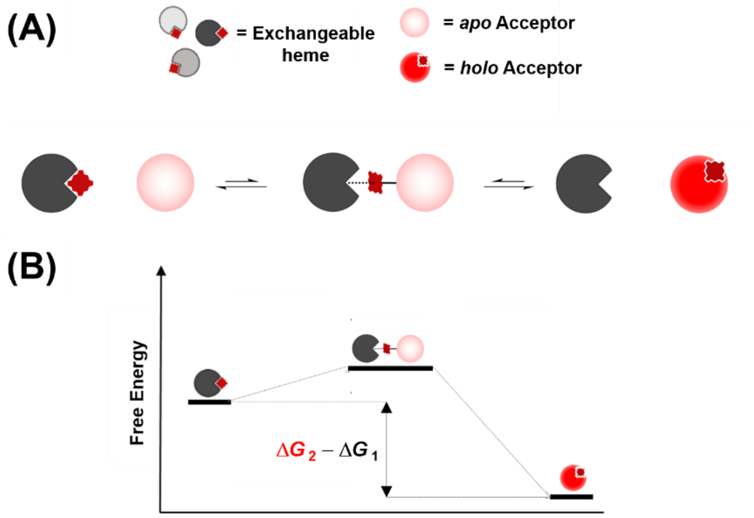

Fig. S7. An alternative exchange mechanism to the example shown in Fig. 4 for managing heme supply and demand in cells based on a classical associative ligand-transfer mechanism.

Parameter	Value	Notes
τ_{S1} (*apo*) / ns	2.7	$\equiv \tau_{Slow}$
τ_{S2} (*apo*) / ns	1.3	$\equiv \tau_{Fast}$
τ_{S1} (*holo*) / ns	1.6	Estimated
τ_{S2} (*holo*) / ns	1.0	Estimated
$k_{Em,S1}$ / s^{-1}	3.7×10^8	Calculated
$k_{Em,S2}$ / s^{-1}	7.7×10^8	Calculated
$k_{RET,S1}$ / s^{-1}	2.5×10^8	Estimated
$k_{RET,S2}$ / s^{-1}	2.5×10^8	Estimated
τ_{Slow} / ns	2.7	Measured
τ_{Fast} / ns	1.3	Measured

Table S1. The photophysical kinetic parameters for purified *apo*- and *holo*-mAPXmEGFP measured in aqueous solution (*in vitro*). Refer to Fig. 1 (B) for an illustration of the decay pathways from the excited states of mAPXmEGFP and definitions of the parameters.

Appendix: Details of the source code for fitting a theoretical single-site binding model to fluorescence decay data.

The fitting program required entry of the concentration of the hemin titrant solution, h_0, the initial volume of the *apo*-protein sample, V_0, and the data points from the titration experiment comprising *n* values for the cumulative addition volume, $\delta V(i)$, and the ratio of the amplitudes for the decay components (either $\alpha_{\text{Fast}}/(\alpha_{\text{Slow}} + \alpha_{\text{Fast}})$ or, if $\alpha_{\text{Slow}} + \alpha_{\text{Fast}} = 1$, then α_{Fast} only). The program also required entry of estimates for the concentration of the protein solution, p_0, and the heme-dissociation constant, K_d, however, these values were refined and optimised by the calculation.

The source code for the simplex algorithm that was used to minimise χ^2 (see equation [9]) was reproduced from (*3*). The subroutine for the downhill simplex method is called *amoeba*, which calls the subroutine called *amotry* to evaluate a trial point, and a customised function to calculate values of χ^2 (the source codes for these are given in (*3*)) The calculation had to be performed using at least three dimensions (*i.e.*, $N = 3$): namely, p_0, K_d, and the value of *C* from equation [8]. Optimised values for these parameters could be obtained by the simplex algorithm. In this case, the number of degrees of freedom, *m*, in equation [9] was equal to $n - 3$, where *n* is the number of data points from the titration experiment. An additional two dimensions, κ and ω, were added to further minimise the value of χ^2. These dimensions enabled account to be taken of the presence of (i) small quantities of heme in the purified protein (ω equals the initial concentration of total heme in the protein sample), and (ii) small amounts of denatured protein which will not bind heme (κ equals the mole fraction of the denatured protein). The addition of these two dimensions in the simplex method requires equations [12], [13] and [14] to be adjusted to:

$$c_{apo}(i) = (1 - \kappa)p_0 \times \frac{V_0}{V_0 + \delta V(i)} - c_{holo}(i) \qquad [19]$$

$$c_{free\ heme}(i) = h_0 \times \frac{\delta V(i)}{V_0 + \delta V(i)} + \omega \times \frac{V_0}{V_0 + \delta V(i)} - c_{holo}(i) \qquad [20]$$

$$0 = c_{holo}(i)^2 - \left(\frac{(1-\kappa)\, p_0 V_0 + h_0 \delta V(i) + \omega \times V_0}{V_0 + \delta V(i)} + K_\mathrm{d}\right) c_{holo}(i) + \frac{(1-\kappa) p_0 V_0 (h_0 \delta V(i) + \omega \times V_0)}{\left(V_0 + \delta V(i)\right)^2} \qquad [21]$$

The increase in the number of dimensions to $N = 5$ leads to a decrease in the value of *m* to $n - 5$.

The downhill simplex method required a set of $N + 1$ vectors of dimension *N* representing different trial values for p_0, K_d, *C*, κ and ω. The different trial values were obtained by starting with initial estimated values, and then making random adjustments to these values in order to obtain sets of $N + 1$ different values. Whilst this was intended to be an unbiased approach, the new values were constrained to a realistic range. A source code for generating random deviates was reproduced from (*3*): the function called *ran1* (minimal standard plus shuffle) generates random numbers between 0 and 1. Random deviates generated by this function were used to transform estimated values for p_0, K_d, *C*, κ and ω into sets of trial values for the simplex method.

The downhill simplex method was most efficient when minimum and maximum possible values for p_0, K_d, *C*, κ and ω were specified. This prevented the propagation of steps in the search for a minimum value of χ^2 in the *N* dimensional volume, where one or more of these quantities cannot be real (for example, the minimum value for each of these quantities must be greater than zero).

The Fortran 90 program is reproduced below with the exception of subroutines and functions from (*3*).

```
      program lifefit
      implicit none

      integer seed,dmax,ITMAX,i,n
      parameter(dmax=100)
      real est_p0,est_Kd,V0,dV(dmax),heme(dmax),afast(dmax),
     + p(6,5),y(6),ftol,h0,limit(4,2)


c   A file needs to be created, input.txt, containing the following data separated by carriage returns:
c        An estimate for the protein concentration, p0, in μM
c        The concentration of the hemin titrant, h0, in μM
c        An estimate for the heme-dissociation constant, Kd, in μM
c        The initial volume of the protein solution, V0, in μL
c        The number of data points, n, from the titration experiment
c        The next n lines must contain two values corresponding to the volume of titrant added, δV,
c            in μL and either αFast/(αSlow + αFast) or, if αSlow + αFast = 1, then  αFast only
c        The tolerance for the minimisation algorithm and the maximum number of allowed iterations
c            [see Ref (3)]
c        Minimum and maximum possible values for p0
c        Minimum and maximum possible values for Kd
c        Minimum and maximum possible values for the constant, C, in equation [8]
c        Maximum possible value for κ in equation [21]
c            (this value must be positive and much less than 1)
c        Maximum possible value for ω in equation [21]
c            (this value must be a small fraction of the maximum possible value for p0)

      open(unit=1,file='input.txt')
        read(1,*) est_p0
        read(1,*) h0
        read(1,*) est_Kd
        read(1,*) V0
        read(1,*) n
        do i=1,n
          read(1,*) dV(i),afast(i)
        end do
        read(1,*) ftol,ITMAX
        read(1,*) limit(1,1),limit(1,2)
        read(1,*) limit(2,1),limit(2,2)
        read(1,*) limit(3,1),limit(3,2)
        read(1,*) limit(4,1)
        read(1,*) limit(4,2)
      close(1)

      do i=1,n
        heme(i)=h0*dV(i)/(dV(i)+V0)
      end do
      seed=-58246
      call create_p(p,est_p0,est_Kd,seed)
      call amoeba(p,y,ftol,dV,heme,afast,n,dmax,V0,limit,ITMAX)
```

```
c  The optimised values for p0, Kd, C, κ and ω are written to a file, output.txt

      open(unit=2,file='output.txt')
        write(2,*) 'protein stock',p(1,1)
        write(2,*) 'Kd',p(1,2)
        write(2,*) 'C',p(1,3)
        write(2,*) 'kappa',p(1,4)
        write(2,*) 'omega',p(1,5)
      close(2)

      end



c   The subroutine create_p generates a set of N + 1 vectors of dimension N representing
c   different trial values for p0, Kd, C, κ and ω

c   Random trial values for the protein concentration, p0, are determined within the range of
c        0.5× to 2.0× the estimated value from the file, input.txt.
c   Random trial values for the heme dissociation constant, Kd, are determined within the range of
c        0.1× to 10× the estimated value from the file, input.txt.
c   Random trial values for the constant, C, in equation [8] are determined within the range of
c        0.5 to 1.5
c   Random trial values for κ are determined within the range of 0 to 0.1
c   Random trial values for ω are determined within the range of 0 to 0.1× the estimated value for
c        p0 from the file, input.txt.

      subroutine create_p(p,est_p0,est_Kd,seed)
      implicit none

      integer seed,i
      real p(6,5),est_p0,est_Kd,ran1

      do i=1,6
         p(i,1)=est_p0*(5.e-1+1.5e0*ran1(seed))
         p(i,2)=est_Kd*(1.e-1+9.9e0*ran1(seed))
         p(i,3)=1.e0*(5.e-1+ran1(seed))
         p(i,4)=1.e-1*ran1(seed)
         p(i,5)=1.e-1*est_p0*ran1(seed)
     end do

     return
     end



c   The function chisq calculates a value for equation [9] and is called by the subroutines amoeba
c   and amotry (from (3)) which are applying the downhill simplex algorithm

      function chisq(ptest,dV,heme,afast,n,dmax,V0,limit)
      implicit none

      integer n,dmax,i
      real dV(dmax),heme(dmax),afast(dmax),ptest(5),p_stock,Kd,Cvalue,
```

```
     + kappa,omega,chisq,total_p,V0,c_holo,f_holo,Fit,limit(4,2)
      p_stock=ptest(1)
      If(p_stock.le.limit(1,1).or.p_stock.gt.limit(1,2))then
        chisq=1.e9
        go to 10
      end if
      Kd=ptest(2)
      If(Kd.le.limit(2,1).or.Kd.gt.limit(2,2))then
        chisq=1.e9
        go to 10
      end if
      Cvalue=ptest(3)
      If(Cvalue.lt.limit(3,1).or.Cvalue.gt.limit(3,2))then
        chisq=1.e9
        go to 10
      end if
      kappa=ptest(4)
      If(kappa.lt.0.e0.or.kappa.gt.limit(4,1))then
        chisq=1.e9
        go to 10
      end if
      omega=ptest(5)
      If(omega.lt.0.e0.or.omega.gt.limit(4,2))then
        chisq=1.e9
        go to 10
      end if

      chisq=0.e0
      do i=1,n
         total_p=p_stock*V0/(V0+dV(i))
         c_holo=((1.e0-kappa)*total_p+heme(i)+omega*V0/(V0+dV(i))+Kd
     +     -SQRT(((1.e0-kappa)*total_p+heme(i)+omega*V0/(V0+dV(i))+Kd)**2
     +    -4.e0*(1.e0-kappa)*total_p*(heme(i)+omega*V0/(V0+dV(i)))))/2.e0
        f_holo=c_holo/total_p
            Fit=(f_holo+Cvalue)/(1.e0+Cvalue)
        chisq=chisq+(afast(i)-Fit)**2
      end do
      chisq=chisq/(real(n-5))

      return
      end
```


1. S. C. Warren *et al.*, Rapid Global Fitting of Large Fluorescence Lifetime Imaging Microscopy Datasets. *Plos One* **8**, e70687 (2013).
2. L. Liu, A. B. Dumbrepatil, A. S. Fleischhacker, E. N. G. Marsh, S. W. Ragsdale, Heme oxygenase-2 is post-translationally regulated by heme occupancy in the catalytic site. *Journal of Biological Chemistry* **295**, 17227-17240 (2020).
3. W. Press, B. Flannery, S. Teukolsky, W. Vetterling, Numerical Recipes in FORTRAN 77: The Art of Scientific Computing. *Cambridge University Press,* 2nd edition (1992).
